# Structural basis of the specific interactions of GRAS family proteins

**DOI:** 10.1002/1873-3468.12987

**Published:** 2018-02-06

**Authors:** Toshio Hakoshima

**Affiliations:** ^1^ Structural Biology Laboratory Nara Institute of Science and Technology Ikoma Japan

**Keywords:** α/β protein, BIRD family, DELLA protein, GRAS domain, IDD family, SAM‐dependent methyltransferase, Transcription cofactor, Transcription factor, Zinc finger

## Abstract

The plant‐specific GAI‐RGA‐and‐SCR (GRAS) family of proteins function as transcriptional regulators and play critical roles in development and signalling. Recent structural studies have shed light on the molecular functions at the structural level. The conserved GRAS domain comprises an α‐helical cap and α/β core subdomains. The α‐helical cap mediates head‐to‐head heterodimerization between SHR and SCR GRAS domains. This type of dimerization is predicted for the NSP1‐NSP2 heterodimer and DELLA proteins such as RGA and SLR1 homodimers. The α/β core subdomain possesses a hydrophobic groove formed by surface α3‐ and α7‐helices and mediates protein–protein interactions. The groove of the SHR GRAS domain accommodates the zinc fingers of JKD, a BIRD/IDD family transcription factor, while the groove of the SCL7 GRAS domain mediates the SCL7 homodimerization.

## Abbreviations

GA, gibberellin

ITC, isothermal titration calorimetry

JA, jasmonate

LHRI, leucine heptad repeats I

OG, orthologous group

ZF, zinc finger

GRAS gene family members were first characterized as being key regulators in plant biology and comprised the three members *GIBBERELLIN‐INSENSITIVE* (*GAI*), Repressor of *ga1‐3* (*RGA*) and *SCARECROW* (*SCR*), together with SCR‐LIKEs (*SCL*s) from a database in 1999 1. Of the initially identified members, the gene products GAI and RGA are members of the DELLA proteins, which are key regulators in gibberellin (GA) signalling and also play important roles in jasmonate (JA) and light signalling 2. SCR acts as a key regulator of radial patterning of Arabidopsis roots together with SHORT‐ROOT (SHR), a member of another class of GRAS proteins 3, 4. At present, 33 and 66 members have been identified as being encoded in Arabidopsis and rice genomes, respectively, and the gene products, GRAS proteins, have since been recognized to play roles in the form of plant‐specific key regulators of transcription in diverse processes including GA signal transduction, phytochrome signalling and root development 2, 5. Another class of GRAS proteins, Nodulation Signalling Pathway proteins regulate nodulation in legumes 2. GRAS proteins function by forming homo‐ or heterodimers and/or interacting with other proteins such as transcription factors 5, 6, 7, 8, 9, 10, 11. Notwithstanding their importance in plant biology, determination of the three‐dimensional structures of GRAS domains had been unsuccessful for quite some time. Recently, two crystallographic structural reports have appeared and clarified the fundamental three‐dimensional structural and physical properties of the GRAS domain 12, 13. One crystallographic report has outlined the structure of the SCR‐SHR heterodimer and its complex with a zinc finger‐type transcription factor of the BIRD/INDETERMINATE DOMAIN (IDD) family, JACKDAW (JKD)/IDD10. SHR and SCR are extensively studied GRAS proteins that act as key regulators in Arabidopsis root development 3, 4. Intriguingly, SHR is referred to as a moving transcriptional regulator since after being transcribed in the stele, it moves into the adjacent layer where SCR sequesters SHR to the nucleus by forming the specific heterodimer SHR‐SCR and blocks SHR movement out of the single cell layer of the endodermis. The SHR‐SCR complex upregulates several genes including zinc finger (ZF) transcription factors of the BIRD/IDD family, containing JKD/IDD10 and MAGPIE (MGP)/IDD3 14, 15. A regulatory network involving BIRD/IDD transcription factors and SCR with SHR organizes tissue patterns at all formative steps during growth, thus ensuring developmental plasticity 16. The other crystallographic report has outlined the structure of rice SCL7 (OsSCL7), which belongs to the SCL4/7 subfamily. Although investigations concerning the biological functions of the SCL4/7 family members are limited, SCL4/7 members may function in response to environmental stress 17. SCL7 is localized in the nucleus, and its overexpression led to increased salt and drought tolerance 18. A recent study indicated that SCL4/7 members also play a role in axillary meristem development 19.

In this review, the available structural information of GRAS domains will be summarized, and consequences of protein–protein interactions including homo‐ and heterodimerization and the recognition of direct targets of GRAS proteins will be addressed in addition to the implications in plant biology.

## GRAS protein classification

**Figure 1:**
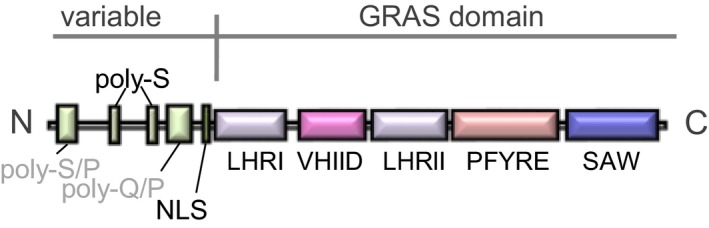
Sequence alignment of GRAS proteins. Domain map of GRAS protein. The domain map of SCR is shown as an example. GRAS domains comprise five designated regions, LHRI, VHIID, LHRII, PYRE and SAW. These historically designated regions do not necessarily correspond to structural/functional subdomains.

Most GRAS proteins comprise an N‐terminal less‐conserved variable region and a C‐terminal conserved GRAS domain. A small number of GRAS proteins, however, have an N‐terminal GRAS domain followed by another functional domain in the C‐terminus, while other members are more exceptional in that they possess double GRAS domains. GRAS genes are usually monoexonic and encode for proteins with lengths between 360 and 850 amino acids. Typical GRAS domains comprise ~ 390 amino acids and can be subdivided into five peptide regions possessing conserved sequence motifs: leucine heptad repeats I (LHRI), VHIID, leucine heptad repeats II (LHRII), PFYRE and SAW 1 (Fig. 1). VHIID, PFYRE and SAW are very short or scattered conserved sequence motifs. As discussed later, each region is found not to form a structural domain but to be part of a conserved structural domain. In contrast with the C‐terminal conserved domain, the N‐terminal region of GRAS proteins appears hypervariable and contains sequences typical of intrinsically disordered proteins 1, 5, 12, 20. Some GRAS proteins contain conserved sequence motifs in the N‐terminal region, which are expected to be involved in molecular recognition. For example, DELLA proteins possess a conserved DELLA sequence motif in the N‐terminal region and the peptide region containing the DELLA motif is found to be conformationally disordered in the free state, but refolds into a helical structure upon binding to GA‐bound GA receptor GID1 20.

Phylogenetic analyses have shown that the GRAS family can be divided into 8–13 subfamilies 1, 21. A very recent study based on a panel of eight representative species of angiosperms (for monocots: *Musa acuminata* (Zingiberales), *Phoenix dactylifera* (Arecales) and *Oryza sativa* (Poales); for dicots: *Arabidopsis thaliana*,* Vitis vinifera*,* Theobroma cacao* (rosids) and *Coffea canephora* (asterid); and *Amborella trichopoda* as the basal angiosperm and outgroup for monocot and dicot phylogenies) identified 29 orthologous groups (OGs) of the GRAS gene family, and these OGs were regrouped into 17 subfamilies (NSP1, SCL32, SHR, PAT1, RAD1, SCLA, SCR, DELLA, RAM1, SCL3, DLT, SCLB, LISCL, SCL4/7, LS, NSP2 and HAM) containing five new subfamilies (DLT, RAD1, RAM1, SCLA and SCLB) (Table 1) 22. Compared with phylogenetic results obtained in a previous study that mainly focused on the model species Arabidopsis and rice, loss of GRAS members within these species was detected in a few OGs (12 and 4 respectively). Although these model species have often been used as a reference, these species have shown evidence of higher evolution rates than others.

**Table 1:** Subfamilies of the GRAS proteins

Subfamily	Functions	Member examples
NSP1	Regulates the expression of nodulation factors, biosynthesis of strigolactone	NSP1
SCL32	Functionally uncharacterized	SCL32
SHR	Root radial patterning and root growth, cell division and endodermis specification transcription factor for nodule development	SHR, SHR1,2
PAT	PhyA‐specific signalling, positive regulator of phyB‐dependent red light signalling hypocotyl elongation, the early stages of plant defence signalling VaPAT1 that confers abiotic stress tolerance in *Arabidopsis thaliana*	PAT1–4, SCL13, CIGR1,2
RAD1	Mycorrhization (missing in all Brassicales)	RAD1
SCLA	Functionally uncharacterized	SCLA
SCR	Root radial patterning and root growth, QC identity, asymmetric cell division	SCR, SCR1–3
DELLA	GA‐response signalling, PIF coactivation, JA signalling modulator	GAI, RGA, RGL1–3, SRL1,2
RAM1	Mycorrhizal signalling (missing in all Brassicales)	RAM1
SCL3	Integrator of GA/DELLA signalling and the SCR/SHR pathway in root cell elongation	SCL3
DLT	Brassinosteroid signalling, its alteration induces dwarf and low‐tillering phenotype in rice	DTL
SCLB	MIG1 is involved in interaction with symbiotic arbuscular mycorrhizal fungi	SCLB
LISCL	The plant stress responses, adventitious root formation in response to auxin	LISCL
SCL4/7	Response to environmental stresses such as salt, osmotic shock and drought	SCL4, SCL7
LS	Formation of lateral shoots during vegetative development	LAS1
NSP2	Regulates the expression of nodulation factors, biosynthesis of strigolactone	NPS2‐1,2,3
HAM	Shoot meristem maintenance, auxin response, nodulation signalling	HAM, SCL1

The most extensively studied subfamilies contain the initially identified SCR, SHR and DELLA protein members. DELLA proteins are key regulators in GA‐response signalling, and are recognized by GA‐bound GID1, which recruits DELLA proteins to the E3 enzyme with F‐box protein SLY for ubiquitylation followed by degradation by the proteasome 2. DELLA proteins also participate in PIF coactivation and function as JA signalling modulators. Arabidopsis DELLA proteins comprise five functional members, GAI, RGA and RGL1‐3, while rice contains one functional DELLA protein SLENDER RICE 1 (SLR1) and two nonfunctional DELLA proteins SLENDER LIKE 1 and 2 (SLRL1 and SLRL2). Although SHR interact with SCR to play a key role in regulating the radial patterning of both the root and shoot 3, 4, they belong to different subfamilies, which are distantly branched in the phylogenetic tree 22. The Arabidopsis SCR subfamily comprises three OGs (SCR1‐3), with SCR and SCL23 being included in these OGs, although OG‐SCR3 is absent. SHR regulates the expression of SCR and SCL23. The SHR subfamily comprises two OGs, SHR1 and SHR2, while SHR2 OG members are absent in both Arabidopsis and rice, and are also absent in the Brassicales and Poales orders.

Since the molecular functions of SCL4/7 subfamily members were poorly understood, the name was derived from two Arabidopsis paralogs SCL4 and SCL7.SCL7 is upregulated under stress conditions, while its close homolog SCL4 is downregulated 17. A *Populus euphratica* ortholog (PeSCL7) was found to be overexpressed during the early stage of induced severe salt‐stress, and transgenic Arabidopsis plants overexpressing this GRAS gene showed increased tolerance to salt and drought stress 18. These experiments suggested that SCL4/7 members participate in response to environmental stress such as salt, osmotic shock and drought.

## Monomer‐dimer equilibrium in solution

**Figure 2:**
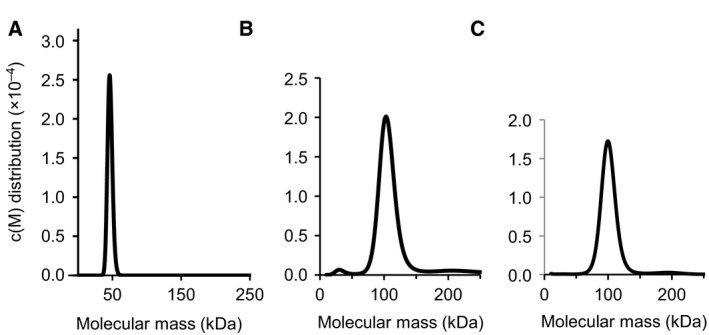
Analytical ultracentrifugation (AUC) of GRAS domains. (A) The SCL5 GRAS domain exists as a monomer. The AUC sedimentation velocity analysis shows an estimated molecular mass of 46.6 ± 3.5 kDa, suggesting a monomer in solution (calculated mass 48.1 kDa). (B) The SCL3 GRAS domain exists as a homodimer. The analysis shows an estimated molecular mass of 106.3 ± 1.6 kDa, suggesting a homodimer in solution (calculated mass 50.8 kDa). (C) The SHR and SCR GRAS domains exist as a heterodimer. The analysis shows a monodispersed state with an estimated molecular mass of 105.4 ± 1.4 kDa, suggesting a heterodimer in solution (calculated mass 89.2 kDa).

Intermolecular interactions between GRAS proteins were analysed using bioassays or biochemical methods such as the yeast two‐hybrid (Y2H) assay, *in vivo* bimolecular fluorescence complementation (BifC) binding assays, or pull‐down binding assays with cell lysates or purified recombinant protein samples 5, 6, 7, 8, 9, 10, 11, 23, 24, 25, 26, 27. The data obtained from such experiments suggested that GRAS proteins could form homodimers and/or heterodimers with other GRAS proteins. For example, SLR1 and *Lotus japonicus* NSP2 were reported to form homodimers 23, 24, while formation of heterodimers was suggested for Arabidopsis SHR‐SCR, SCL3‐RGA and SCL3‐GAI, *Medicago truncatula* NSP1‐NSP2 and RAM1‐NSP2 and *L. japonicus* RAD1‐RAM1 and RAD1‐NSP2 8, 11, 23, 24, 25, 26, 27. RAD1 did not interact with itself, suggesting that RAD1 may exist as a monomer in the absence of RAM1 and NSP2. More definitive evidence showing oligomerization of GRAS proteins has been obtained using hydrodynamic analyses such as analytical ultracentrifugation (AUC) with purified protein samples (Fig. 2) 12. The analyses using AUC clarified the stable monodispersed states of Arabidopsis GRAS proteins in solution with estimated molecular masses showing that SCL5 exists as a monomer, while SCL3 exists as a homodimer, and SHR‐SCR exist as a 1 : 1 heterodimer. Interestingly, the AUC profiles show a single dominant peak without additional major peaks, suggesting that each dimeric or monomeric form would be stable in solution, rather than as two or more multiple metastable forms in equilibrium.

In efforts to identify subdomains and/or motifs that are responsible for the dimerization, LHRI and LHRII regions were repeatedly deduced to be critical regions for homo‐ and heterodimerization 8, 11, 23, 27. The Y2H assay showed that the LHRI‐VHIID‐LHRII region is important for SCR‐SHR binding 8. The LHRI region of NSP2 is essential for the NSP1–NSP2 interaction and the LHRI region of RAD1 is essential for the RAD1–RAM1 interaction 11, 27. Similar dependencies were observed for DELLA protein members: the LZ region, which corresponds to the LHRI region, of SLR1 is important for homodimerization 23. These results are consistent with the obtained structure, where the LHRI region was found to be directly involved in the intermolecular interactions in the SHR‐SCR dimer 12 (see below).

## GRAS domain structure

**Figure 3:**
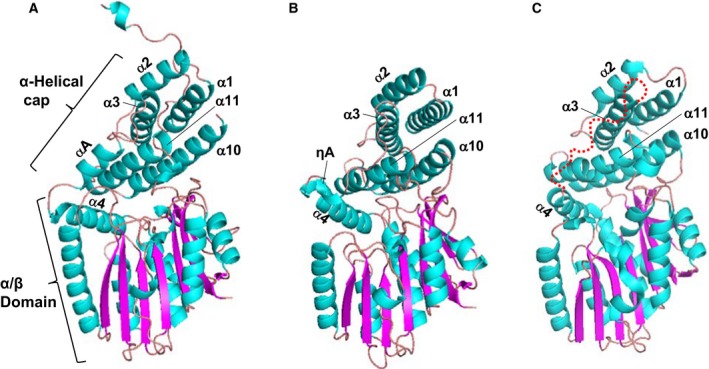
GRAS domain structure. (A) the GRAS domain structure of SHR. The GRAS domain comprises an α‐helical cap and α/β core subdomains. The colour codes are cyan for α‐ and 3_10_‐helices and magenta for β‐strands. (B) As in (A), but for the GRAS domain structure of SCR. (C) As in (A), but for the GRAS domain structure of SCL7. The current SCL7 structure lacks the peptide segment (a red dotted line) linking for α3‐ and α4‐helices. The segment of SHR forms αA‐helix and that of SCR forms ηA‐helix in the SHR‐SCR complex (see A,B).

**Figure 4:**
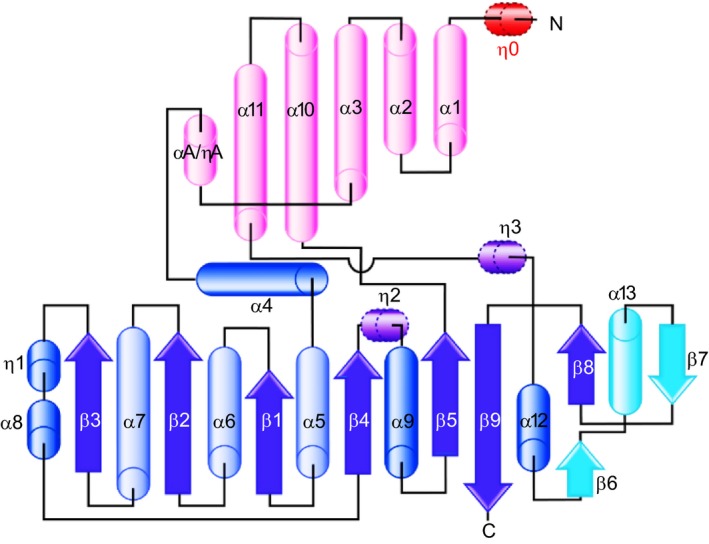
Topology of GRAS domain. GRAS domain comprises α‐helical cap (pink) and α/β core (blue and cyan). The SHR GRAS domain contains fourteen α‐helices (α1–α13 and αA), four 3_10_‐helices (η0–η3) and nine β‐strands (β1–β9). Compared with the α/β folds of SAM‐MTs, the α/β core of the GRAS domain possesses an additional β6‐α13‐β7 segment (cyan).

The reported crystal structures of the SHR‐SCR heterodimer and the SCL7 homodimer were determined at 2.0 and 1.8 Å resolution, respectively 12, 13. These structures revealed a common subdomain organization of the GRAS domain comprising an α‐helical cap and α/β core subdomains (Fig. 3). The SHR GRAS domain contains fourteen α‐helices, three 3_10_ helices and nine β‐strands, which form a central β‐sheet (Fig. 4). The architecture belongs to the α/β folds of *S*‐adenosyl methionine‐dependent methyltransferases (SAM‐MTs). The central β‐sheet of SAM‐MT comprises seven β‐strands, which are conserved in the GRAS domain. In addition to the seven‐stranded β‐sheet, GRAS domains have two additional strands (β6 and β7) and one α‐helix (α13) forming a β6‐α13‐β7 segment at one edge of the central β‐sheet. This segment is important for protein–protein interactions that mediate direct binding between SHR and the zinc finger of the BIRD/IDD family of transcription factors, or to mediate dimerization of SCL7 (see below).

The α‐helical cap of SHR forms an antiparallel helix bundle comprising six helices: three N‐terminal α‐helices (α1, α2 and α3) encompassing the LHRI motif, two α‐helices (α10 and α11) from the α/β core subdomain, and αA‐helix, which is located between α3 and α4 helices and links the α‐helical cap and α/β core subdomains (Fig. 3). SCR has a short 3_10_‐helix (ηA) and a loop *in lieu* of the αA‐helix of SHR. In SCL7, a helix corresponding to αA‐helix of SHR or ηA‐helix of SCR was not found, and the entire loop between α3 and α4 helices is invisible probably due to a disordered structure. The α‐helical cap sits on the α/β core subdomain, which incorporates a nine‐stranded mixed β‐sheet at the centre with seven α‐helices on both sides of the β‐sheet. The main part of the central β‐sheet, β3‐β2‐β1‐β4‐β5, is parallel, but the remaining part of the β‐sheet, β9‐β8/β6‐β7, is antiparallel. One side contains two α‐helices (α9 and α12) and a long loop containing a short α‐helix (α8) and a short 3_10_‐helix (η1), whereas the other side contains helices (α4, α5, α6, α7 and α13) forming a large groove, which mediates protein–protein interactions as shown below. Among the five conserved sequence motifs characterized in the absence of structural aspects, only the LHRI motif corresponds to a structural unit, part of the α‐helical cap as described above, while others (VHIID, LHRII, PFYRE and SAW) are component parts of the α/β core subdomain.

The α‐helical caps of SHR and SCR show a similar structure with small root‐mean square (rms) deviation (1.23 Å) of Cα‐carbon atom positions following structural superimposition. The structures of the α/β core subdomains also show high similarity with small rms deviation (1.69 Å). The overall structures of the GRAS domains, however, display a somewhat large deviation (2.7 Å). This discrepancy is caused by a movement of the α‐helical cap with respect to the α/β core subdomain. The well‐conserved subdomain structures of the α‐helical cap and α/β core subdomains are observed in the SCL7 structure. The overall structure of the SCL7 GRAS domain resembles that of the SHR structure (2.0 Å) rather than that of SCR (2.3 Å).

**Figure 5:**
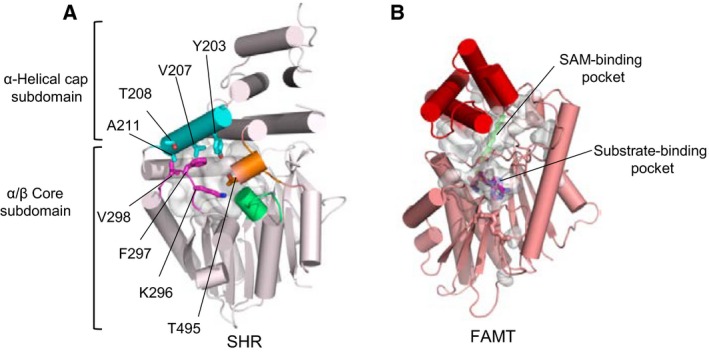
Cavity of the GRAS domains. (A) The SHR GRAS domain has a large cavity (grey). The cavity of the SHR GRAS domain is located at the central region enclosed by αA helix (cyan), β2–α7 loop (magenta), β4–α9 loop (green) and α11–α12 loop (orange), and occupies a large internal space (1529 Å^3^). (B) The active site cavity of *Mycobacterium marinum* fatty acid *O*‐methyltransferase (FAMT), a member of the SAM‐MT family. The cavity contains bound SAH (magenta) and substrate (green) molecules in the cavity (1309 Å^3^).

GRAS domains possess a large cavity in the α/β core covered by the α‐helical cap, as observed in members of the SAM‐MT family (Fig. 5). However, GRAS domains lack the SAM‐binding motifs, which are conserved in SAM‐MT members and no binding was observed for SAM, *S*‐adenosyl homocysteine, or the product monomethyl‐L‐lysine in our binding assays using isothermal titration calorimetry (ITC) 12, suggesting that the GRAS domains lack methyltransferase activity.

## Structures of GRAS domain dimers

**Figure 6:**
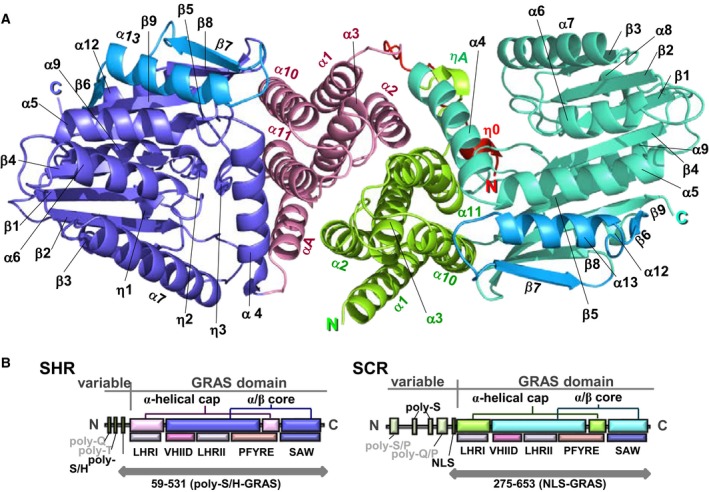
GRAS domain heterodimer, SHR‐SCR. (A) Ribbon diagram of the Arabidopsis SHR‐SCR GRAS domain heterodimer. SHR comprises the N‐terminal strap (in red), α‐helical cap (magenta) and α/β core (blue) subdomain; SCR consists of the α‐helical cap (green) and α/β core (turquoise). The α/β core subdomains contain GRAS‐specific segments, β6–α13–β7(cyan in SHR and light blue in SCR). (B) Domain and motif organizations of SHR and SCR GRAS domains.

The GRAS domains of SHR and SCR form a 1 : 1 heterodimer with pseudo‐dyad symmetry 12 (Fig. 6). Dimerization of SHR and SCR GRAS domains is mediated by the α‐helical caps to form a head‐to‐head dimer. The dimer interface comprises eight α‐helices (α2, α3, αA/ηA and α11) and four loops (α2‐α3 and α10‐α11) from both α‐helical caps with a large buried accessible surface area (~ 2070 Å^2^). Both polar and nonpolar contacts comprise the interface, involving direct hydrogen bonding and salt bridging, water‐mediated hydrogen bonding, and hydrophobic interactions. The interface contains asymmetric interactions, which may confer specificity required for SHR‐SCR heterodimerization. Notably, the SCR nonpolar segment encompassing the C‐terminal half of α3 helix followed by α3‐ηA loop and short 3_10_‐helix ηA acts as a ‘hydrophobic belt’, which wraps around α2 helix from SHR with nonpolar contacts. The hydrophobic belt is conserved in SCR beyond species but not in other GRAS proteins, and nonpolar residues of SHR α2 helix are also conserved and specific for SHR. In SHR, most of the long α3‐ηA loop is folded into the αA helix, which makes a parallel helix‐helix interaction with α2 helix from SCR. Thus, these interactions should be specific to heterodimerization between SHR and SCR.

**Figure 7:**
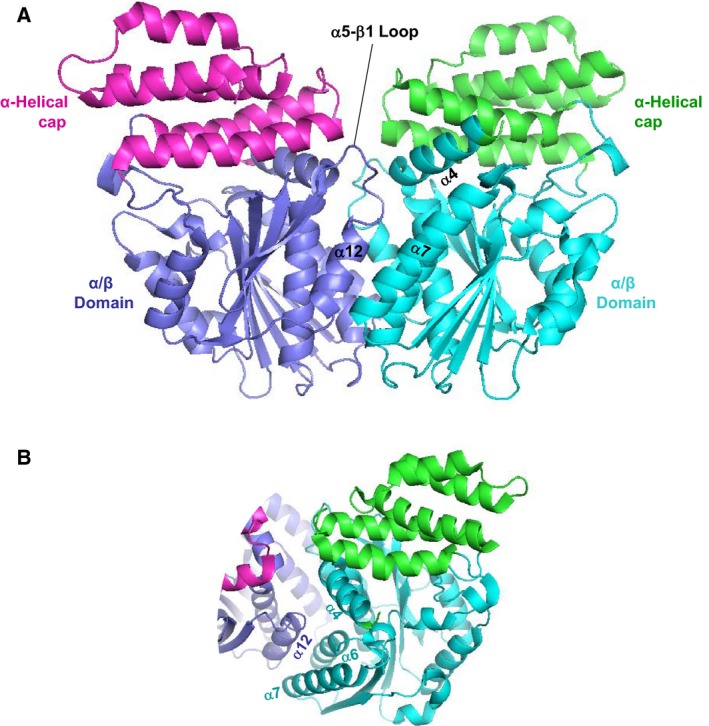
GRAS domain homodimer, SCL7‐SCL7. (A) Ribbon diagram of the rice SCL7 GRAS domain homodimer. The GRAS domain comprises the α‐helical cap (magenta/green) and α/β core (blue/cyan). The homodimer is formed with the contact between the α/β core subdomains. (B) The dimer interface comprises α4‐ and α7‐helices forming a groove that accommodates α12 helix from the other protomer. At the groove, α12 helix primarily contacts with α6‐ and α7‐helices.

The SHR‐SCR heterodimeric structure naturally suggests that a class of GRAS domains may be capable of forming dimers mediated by the antiparallel helix bundle of the α‐helical cap. However, the homodimeric structure of the SCL7 GRAS domain provides another variation in dimerization (Fig. 7) 13. The SCL7 GRAS domain forms a dimer in which protomers are related by crystallographic dyad symmetry. The interface consists of a groove formed by α4‐ and α7‐helices at the molecular surface of the α/β core subdomain and α12 helix from the other protomer docked into the groove. The interface produces a buried accessible surface area (1346 Å^2^), whereas this area is significantly smaller than that of the SHR‐SCR heterodimer. Consistently, SCL7 exists in an equilibrium between monomeric and dimeric states in solution, as shown by size exclusion chromatography (SEC) analysis 13. The α‐helical cap of SCL7 has an abnormal feature in that nonpolar sidechains of three alanine residues (Ala227, Ala230 and Ala234) from α2‐helix, Phe248 and Ala259 from α3‐helix and Trp489 from α11‐helix are exposed to the solvent region. These exposed nonpolar residues form a hydrophobic patch on the molecular surface and suggest that the α‐helical cap of SCL7 should have a hitherto unidentified binding partner.

## Downstream effector recognition by GRAS domains

**Figure 8:**
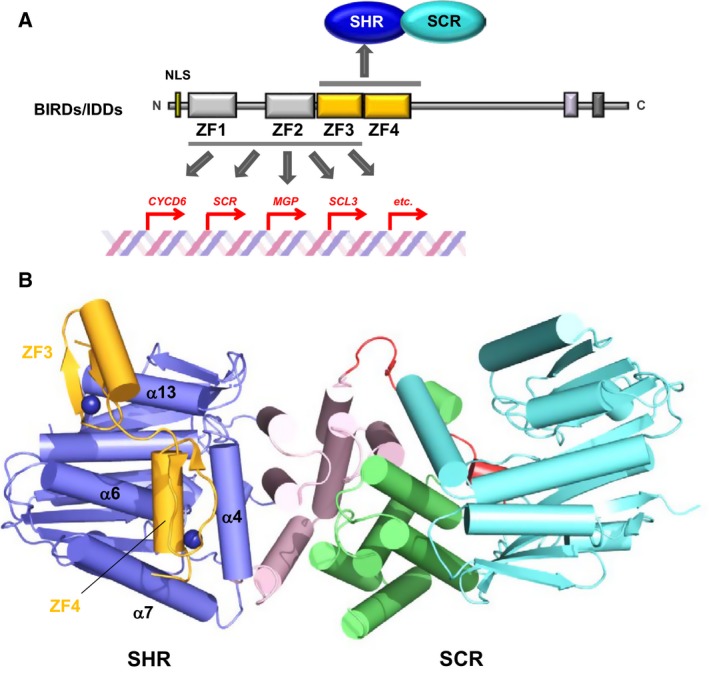
BIRD/IDD recognition by the SHR‐SCR heterodimer. (A) BIRD/IDD transcription factors contain four conserved zinc fingers (ZFs) at the N‐terminal regions, with the first two (ZF1 and ZF2) of the C_2_H_2_ type ZF and the next two (ZF3 and ZF4) of the C_2_ HC type. ZF1–3 mediates specific binding to DNAs of target genes. (B) The structure of the JKD‐SHR‐SCR complex. The SHR GRAS domain comprises the helical cap (magenta) and the α/β core (blue) and also the SCR GRAS domain comprises the helical cap (green) and the α/β core (cyan). The JKD ZF3 and ZF4 modules (orange) directly bound to SHR of the SHR‐SCR heterodimer. The groove formed by α4‐ and α7‐helies of the α/β core accommodates the ZF4 α‐helix.

The interaction between the SHR‐SCR complex and BIRD/IDD transcription factors is one of the best studied examples of target effector protein recognition by GRAS proteins. BIRD/IDD transcription factors contain four conserved zinc fingers (ZF1–ZF4) at the N‐terminal region and ZF3–ZF4 is important for binding to the SHR‐SCR complex (Fig. 8). ITC experiments identified the relatively high affinity of the SHR‐SCR complex to MGP/IDD3 ZF3‐ZF4 (*K*_D_ = 36 nm) and JKD/IDD10 ZF3‐ZF4 (*K*_D_ = 124 nm) 13. The crystal structure of the SCR‐SHR heterodimer bound to ZF3‐ZF4 of JKD, the JKD‐SHR‐SCR complex structure, has been determined at 2.7 Å resolution 13. The crystal structure reveals that the zinc fingers ZF3 and ZF4 bind directly to SHR of the SHR‐SCR complex (Fig. 8). Each ZF of JKD possesses a common ββα‐type structure representative of a classical C_2_HC zinc‐finger. It is well‐established that the α‐helix of ββα‐type ZFs docks into the major groove of DNA for reading of the DNA sequence 28. In the JKD‐SHR‐SCR complex structure, the α‐helix of ZF4 is docked into the groove formed by α4‐ and α7‐helices of the α/β core subdomain of SHR with stabilization by nonpolar interactions, while the β‐sheet of ZF3 binds a shallow groove formed by α13‐ and α6‐helices of the α/β core subdomain and is stabilized *via* polar interactions. Thus, the orientation of the two ZFs of JKD against SHR differ and the α‐helix of ZF3 is accessible to DNA, but not that of ZF4. The observed binding mode is consistent with the fact that unlike the case with ZF4, zinc fingers ZF1‐ZF3 are critical for DNA binding.

**Figure 9:**
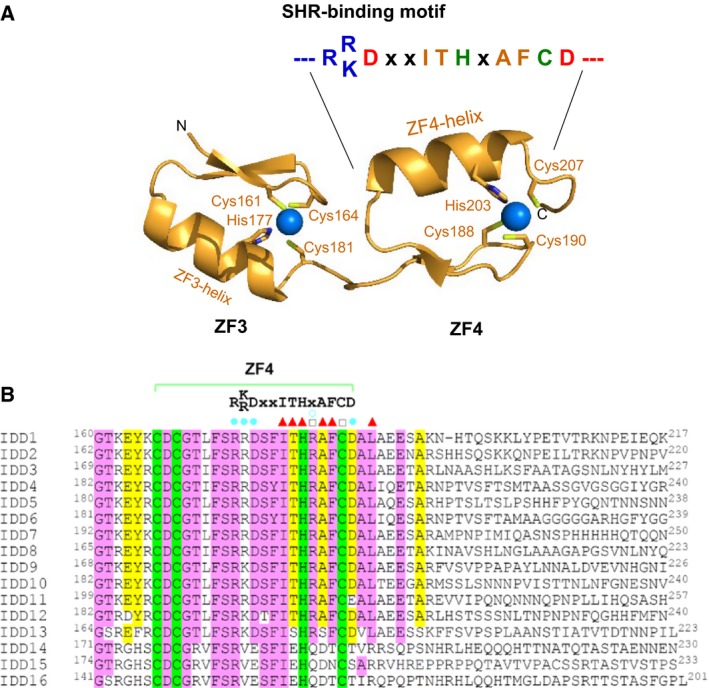
The SHR‐binding motif. (A) The SHR‐binding motif found in the α‐helix of ZF4 of JKD. The conserved residues IT and AF are located the nearly the same helix surface forming the interface with SHR on docking into the SHR groove. (B) Sequential alignment of the ZF4 zinc fingers among IDD family proteins. The SHR‐binding motif is conserved in 13 members (IDD1–13) of the BIRD/IDD family of transcription factors. Sequence alignment of the ZF4 zinc fingers in *Arabidopsis thaliana* IDDs. Highly conserved residues (more than 80%) and relatively conserved residues (60–80%) are filled in pink and yellow, respectively, while the conserved Cys or His residues which are essential for coordinating zinc ions are filled in green. In the JKD‐SHR‐SCR ternary complex 12, JKD/IDD10 residues whose side chain atoms form hydrogen bonds with SHR residues, residues whose main chain atoms form hydrogen bonds with SHR residues and residues involved in hydrophobic interactions with SHR are marked with filled circle (cyan), open circle (cyan) and triangle (red), respectively.

The ZF4 α‐helix bound to the SHR groove contains the SHR‐binding motif that comprises the ZF4‐specific sequence R(K/R)DxxITHxAFCD (in which x represents any amino acid residue). The SHR‐binding motif is highly conserved in 13 (from IDD1 to IDD13) of the 16 members of the *A. thaliana* BIRD/IDD family of transcription factors (Fig. 9). The other three members, IDD14, IDD15 and IDD16, lack a Phe residue corresponding to Phe206 of JKD ZF4, and lack other residues important for SHR binding. The SHR‐binding motif is less conserved in other GRAS proteins, suggesting specific binding to SHR but not to other GRAS family proteins.

**Figure 10:**
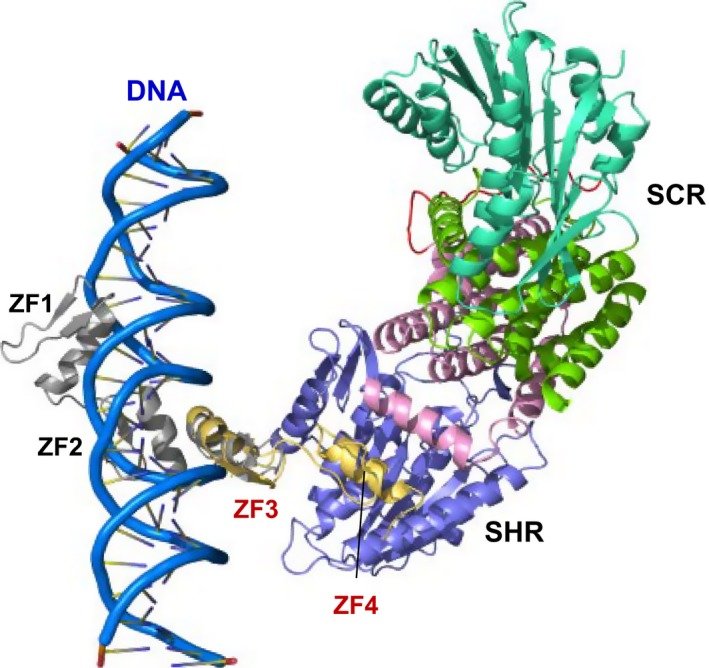
The BIRD/IDD‐SHR‐SCR bound to DNA. A model of the JKD‐SHR‐SCR complex bound to DNA. The JKD ZF1–3 bound to DNA to read out the sequence and the ZF3 and ZF4 bound to SHR of the SHR‐SCR complex.

The crystal structure of the DNA‐bound form of mouse immediate early protein Zif268, which is a typical ZF transcription factor containing three tandem repeats of zinc fingers, revealed that each ZF is folded into a typical ββα structural module, which is docked into the major groove of DNA in a configuration where the ZF α‐helix is inside the DNA groove and the β‐sheet is outside of the groove 29. This binding mode enabled us to build a model of the DNA‐bound JKD‐SHR‐SCR complex (Fig. 10). The model suggests that SHR‐SCR are transcriptional cofactors that regulate target gene transcription *via* binding of SHR to BIRD transcription factors.

In contrast with the JKD‐SHR‐SCR complex structure, the SCL7 homodimer was suggested to bind directly to DNA 3. This idea was drawn from the fact that the SCL7 homodimer found in the crystal has a positively charged cleft between the SCL7 protomers. If the cleft is expanded by 6.4 Å in width, this enlarged cleft has been shown to be capable of accommodating a double‐stranded DNA helix. *In vitro* binding analysis using electrophoretic mobility shift assays with an oligonucleotide having a modelled sequence and purified recombinant SCL7 GRAS domain showed a band shift indicating formation of a protein–DNA complex, although the band shift was rather faint, probably being partly caused by using an oligonucleotide with an artificial sequence. Further experiments are required to confirm the DNA binding activity of the GRAS domain by identifying the genuine target DNA sequence(s) involved.

## General aspects of GRAS protein interactors inferred from crystal structures

Based on the reported structures, it is clear that the GRAS domain possesses at least two active sites for protein–protein or protein–ligand interactions. One site is the α‐helical cap, which facilitates intermolecular helix‐bundle formation to form a GRAS domain dimer. The SCH‐SCR heterodimer is an example of α‐helical cap‐mediated dimerization and similar dimerization modes mediated by the α‐helical cap are predicted in homodimers of other GRAS proteins. For example, α‐helical cap‐mediated dimerization of RGA is expected based on the results of interaction site mapping that showed impaired RGA homodimerization by deletion of the LHRI region (the LRI region) 30. Impaired homodimerization by deletion of the LZ region, which encompasses LHRI, supports SLR1 dimerization mediated by the α‐helical cap 23. Moreover, the importance of the LHRI region in heterodimerization of *M. truncatula* NSP1 and NSP2 has also been demonstrated 26. The interaction between the α‐helical caps of SHR and SCR is mediated by nonpolar contacts in addition to polar contacts. Therefore, in the absence of binding partners, the hydrophobic patch formed by surface nonpolar residues should be exposed to the solvent region and may destabilize the α‐helical cap structure or tend to facilitate the formation of nonspecific aggregates. Moreover, the N‐terminal variable region directly linked to the α‐helical cap is characterized by conformationally flexible sequences displaying no stable secondary structure in the absence of binding partners. These facts partly account for the reason why the preparation of recombinant protein samples of GRAS proteins for structural and physical studies is relatively difficult compared with other soluble proteins. Studies directed towards identifying binding partners, which are other GRAS proteins or may be other classes of proteins, are essential and structural studies of their respective stable complex forms may overcome the difficulty associated with structural studies of isolated GRAS proteins. The crystal structure of SCL7 shows a homodimeric form with the free state of the α‐helical cap, while the α‐helical cap possesses surface hydrophobic patches that are capable of mediating interactions with other molecules. Thus, the presence of surface hydrophobic patches on the α‐helical cap seems to be common to all GRAS domains and contribute to intermolecular interactions.

The other potential site for intermolecular interactions is the hydrophobic groove formed by α3‐ and α7‐helices on the α/β core of GRAS domains. This groove is found in all three GRAS domain structures of SHR, SCR and SCL7, suggesting the presence of common structural characteristics in GRAS domains. The zinc‐finger ZF4 of the JKD transcription factor of the BIRD/IDD family binds this hydrophobic groove of the SHR α/β core subdomain in the JKD‐SHR‐SCR structure. Detailed inspection of the binding mode revealed the SHR‐binding motif found in the ZF4 zinc finger. Intriguingly, 13 (IDD1‐IDD13) of the 16 members of the BIRD/IDD family have a conserved SHR‐binding motif, predicting that these 13 transcription factors function in conjunction with SHR. Since the 13 transcription factors bind a common binding site, the SHR‐binding of these transcription factors is exclusive. This type of binding competition could play a key role in regulation, as seen with JKD and MGP 12. Thus, structural information of protein‐protein complexes provides valuable information for understanding biologically important proteins. The hydrophobic groove formed by α3‐ and α7‐helices is extended to a negatively charged shallow groove formed by α13‐ and α6‐helices. This groove accommodates the positively charged β‐sheet of the ZF3 zinc finger of JKD in a fashion enabling this zinc finger to bind DNA. Our model building shows that JKD can bind DNA in the SHR‐SCR‐bound form, suggesting that SHR‐SCR plays a role as a transcriptional cofactor, which does not bind DNA directly but interacts with other transcription factors or general transcription factors such as polymerases. SCR possesses a hydrophobic groove formed by α3‐ and α7‐helices, although the binding partner is unknown. SCR also possesses a shallow groove formed by α13‐ and α6‐helices, but this groove has no negative charges, suggesting distinct specificity from that of SHR.

SCL7 also possesses a hydrophobic groove formed by α3‐ and α7‐helices. In this case, the groove accommodates α12‐helix (corresponds to α13‐helix of SHR) from the other protomer to mediate homodimerization by α/β core subdomain docking with direct interactions with α7‐helix and α6‐helix at the bottom of the groove. Thus, the hydrophobic groove may be utilized for dimerization of some GRAS proteins. A positively charged putative DNA‐binding groove is located between the SCL7 protomers, suggesting that some GRAS proteins may function as transcription factors that bind directly to DNA. However, additional experimental evidence is required to confirm this possibility. Direct DNA‐binding of *M. truncatula* NSP1 has been demonstrated with identification of the core AATTT motif in the target ENOD11, NIN and ERN1 promoters 26. A closer relationship or common sequence characteristics between NSP1 and SCL7 might be expected, whereas SCL7 shows low sequence identity (14%) with NSP1. Moreover, the α‐helical cap but not the α/β core subdomain was shown to be essential for dimerization of NSP1 and NSP2, as described above. These results suggest that the DNA‐binding model of NSP1 and NSP1‐NSP2 should differ from the model proposed for SCL7 13. The SHR‐SCR heterodimer shows that both the SHR and SCR GRAS domains are overall negatively charged and lack the prerequisite for direct DNA‐binding.

## Other GRAS protein interactors

Binding partners of DELLA proteins have been extensively investigated and a dozen direct binding proteins have been reported 31. The DELLA‐interacting proteins include basic helix‐loop‐helix (bHLH) type transcription factors such as PHYTOCHROME‐INTERACTING FACTORs (PIFs), SPATURA (SPT) and ALCATRAZ (ALC), and other classes of transcription factors, such as BRASSINAZOLE RESISTANT1 (BZR1)/BES1, ETHYLENE INSENSITIVE 3 (EIN3), and SQUAMOSA PROMOTERBINDING PROTEIN‐LIKEs (SPLs). DELLA binding facilitates sequestration of these transcription factors from direct binding to promoter DNA. DELLA interacts with JASMONATE ZIM‐DOMAIN (JAZ), which is a negative regulator of JA signalling, and binds the MYC2 bHLH transcription factor to suppress its transcriptional activity 32, 33. This interaction relieves the JAZ‐mediated repression of MYC2 transcriptional activity. All these interactions are thought to be mediated by the GRAS domains, although the binding modes remain unknown.

BZR1 and PIFs cooperatively function to integrate brassinosteroid and environmental responses by binding to each other's DNA‐binding domains 30, 34. DELLA proteins such as RGA inhibit transcriptional activity by sequestration of these transcription factors from direct binding to promoter DNA. Recently, DELLA proteins have been shown to be glycosylated at the N‐terminal DELLA regions by O‐GlucNAc transferase SECRET AGENT (SEC) and O‐fucosyltransferase SPINDLY (SPY) 35. Intriguingly, O‐fucosylation and O‐GlucNAcylation of RGA modulate the interaction with BZR1 and PIFs: O‐fucosylation accelerates while O‐GlucNAcylation represses RGA binding to BZR1 and PIFs. A model of conformational switching between open and closed states, which could be mediated by covering/uncovering of the GRAS domain with the DELLA region, has been proposed. Further structural studies of the two states of GRA are expected to shed light on the mechanism by which RGA functions as a conformational switch to regulate the integration of multiple signals.

A Y2H assay showed that RGA and SCL3 bind members (IDD3, 4, 5, 9, 10) of the BIRD/IDD family of transcription factors in Arabidopsis 36. These IDD‐DELLA and ‐SCL3 interactions competitively regulate downstream gene expression: DELLA binding upregulates while SCL3 binding downregulates target gene expression such as *SCL3*. Unexpectedly, binding‐site mapping showed that the GRAS domains bind the C‐terminal region of IDD3/MGP, but not the zinc fingers. A similar result was also reported for the interaction between GAI and GAI‐ASSOCIATED FACTOR 1 (GAF1), which is a zinc finger transcription factor closely related to IDD1 37. The GRAS domain of GAI binds the C‐terminal region of GAF1 and upregulates target gene expression. Although the binding mode between the GRAS domains and IDD3/MGP or the GAF1 C‐terminal region remains to be clarified, this fact suggests that the BIRD/IDD family of transcription factors possess two binding sites for different GRAS proteins and function to integrate different signals. Structural studies of the interplay of multiple proteins that upregulate or downregulate target gene expression can provide a powerful clue towards our understanding of the complexity and multiplicity of gene regulation of plants.
